# Supplementary material for: Integrating chromatin conformation information in a self-supervised learning model improves metagenome binning
Source: PeerJ. 2023 Sep 22;11:e16129. doi: 10.7717/peerj.16129 (PMC10519199; doi:10.7717/peerj.16129)
Supplement: Supplemental Information 1 [file peerj-11-16129-s001.pdf]

Figure S1

Hi-C sequencing library parameters

Library	Organism	Cell Numbers	Library Type	Kit	Kit Version
GBBOG	ZymoBIOMICS™ Microbial Community DNA Standard: D6305	100ng DNA	Shotgun	Kapa Hyper Prep Kit	N/A
CZWOH	ZymoBIOMICS™ Microbial Community Standard cells: D6300	1E8 cells	Hi-C	Phase Genomics	1
CZWON	ZymoBIOMICS™ Microbial Community Standard cells: D6300	2.8E8 cells	Hi-C	Arima/Kapa	1
GPANX	ZymoBIOMICS™ Microbial Community Standard cells: D6300	2.8E8 cells	Hi-C	Arima/Kapa	1
GPANY	ZymoBIOMICS™ Microbial Community Standard cells: D6300	1E8 cells	Hi-C	Phase Genomics	1.5
GPANZ	ZymoBIOMICS™ Microbial Community Standard cells: D6300	1E8 cells	Hi-C	Phase Genomics	1.5
GPAOA	ZymoBIOMICS™ Microbial Community Standard cells: D6300	1E8 cells	Hi-C	Phase Genomics	1.5
GPAOB	ZymoBIOMICS™ Microbial Community Standard cells: D6300	1E8 cells	Hi-C	Phase Genomics	1.5

Figure S2

Commands-line options of tools used

Quality and adapter filtering

bbduk.sh in=<wgs.fastq> out=<adapter_filtered_wgs_1.fastq> out2=<adapter_filtered_wgs_2.fastq> k=23 ktrim=r mink=12 hdist=1 minlength=50 tpe tbo

bbduk.sh in=<adapter_filtered_wgs_1.fastq> in2=$<adapter_filtered_wgs_2.fastq> out=<quality_filtered_wgs_1.fastq> out2=<quality_filtered_wgs_2.fastq> qtrim=rl trimq=10 minlength=50 chastityfilter=True

Metagenome assembly and initial binning

metaspades.py -m 1500 -t 60 -1 <quality_filtered_wgs_1.fastq> -2 <quality_filtered_wgs_2.fastq> -o <spades_output>

bbmap.sh in<quality_filtered_wgs_1.fastq> in2=<quality_filtered_wgs_2.fastq> ref=<scaffolds.fasta> out=<wgs_bbmaped.bam>

samtools sort <wgs_bbmaped.bam> -o <wgs_sorted_bbmaped.bam>

samtools index <wgs_sorted_bbmaped.bam>

runMetaBat.sh -m 1500 <scaffolds.fasta> <wgs_sorted_bbmaped.bam>

Running MetaBAT-LR

Metabat_LR.sh -s <scaffolds.fasta> -1 <hic_1.fastq> -2 <hic_2.fastq> -b <input_bins_directory> -d <depth_file.txt> -t <threads> -o <output_directory>

Running bin3C

bwa mem -5SP -t <threads> <contigs.fasta> <hic_1.fastq> <hic_2.fastq> | \
  samtools view -F 0x904 -bS - | \

samtools sort -n -o <mapped.bam> -

bin3C.py mkmap -e <enzyme1> -e <enzyme2> -v <contigs.fasta> <mapped.bam> <bin3c_out_directory>

bin3C.py cluster -v <contact_map.p.gz> <clust_out_directory>

Running HicSPAdes
hicspades-binner <assembly_graph.gfa> <dataset_description.yaml> <output_directory>

Running BinSPreader
bin-refine <assembly_graph.gfa> <initial_binning_file.tsv> <output directory>

Running dRep
dRep dereplicate <output_directory> -p <threads> -d -l 200000 -sa 0.97 -comp 5 -con 99 -g <genome_input_list.txt>

python3 amber.py -g <gold_standard.txt> <method1_bins_biobox.tsv> <method2_bins_biobox.tsv> < method3_bins_biobox.tsv > -o <amber_output>

Figure S3

Quast output data of Synthetic Dataset

Total Aligned Length of Synthetic Metagenome Bins Before MetaBAT-LR was Applied (in bp)

Reference Genomes	bin.1	bin.10	bin.11	bin.12	bin.13	bin.14	bin.15	bin.2	bin.3	bin.4	bin.5	bin.6	bin.7	bin.8	bin.9
Bacillus subtilis	-	-	-	-	-	-	-	-	1818749	-	1135237	-	890877	-	-
Cryptococcus neoformans	-	-	-	-	-	-	1927	-	-	-	-	-	-	-	-
Enterococcus faecalis	-	-	-	1117655	-	-	-	-	74	1459877	-	-	-	-	-
Escherichia coli	-	-	-	-	-	4538063	-	-	-	-	-	-	-	-	-
Lactobacillus fermentum	-	266607	236079	-	-	-	-	-	-	-	-	497487	-	-	374862
Listeria monocytogenes	-	-	-	-	-	-	-	-	74	-	-	-	-	2922858	-
Pseudomonas aeruginosa	-	-	-	-	6689753	-	-	-	-	-	-	-	-	-	-
Saccharomyces cerevisiae	-	-	-	-	-	-	4712838	-	-	-	-	-	-	-	-
Salmonella enterica	3801292	-	-	-	-	814409	-	-	-	-	-	-	-	-	-
Staphylococcus aureus	-	-	-	-	-	-	-	2636514	79	-	-	-	-	-	-

Total Aligned Length of Synthetic Metagenome Bins After MetaBAT-LR was Applied (in bp)

Reference Genomes	bin.1	bin.10.11.9.6	bin.12.4	bin.13	bin.14	bin.15	bin.2	bin.3.7.5	bin.8
Bacillus subtilis	-	-	-	-	-	-	-	3844863	-
Cryptococcus neoformans	-	-	-	-	-	8840412	-	-	-
Enterococcus faecalis	-	-	2577532	-	-	-	-	74	-
Escherichia coli	-	-	-	-	4538063	-	-	-	-
Lactobacillus fermentum	-	1375035	-	-	-	-	-	-	-
Listeria monocytogenes	-	-	-	-	-	-	-	74	2922858
Pseudomonas aeruginosa	-	-	-	6689753	-	-	-	-	-
Saccharomyces cerevisiae	-	-	-	-	-	9546496	-	-	-
Salmonella enterica	3801292	-	-	-	814955	-	-	-	-
Staphylococcus aureus	-	-	-	-	-	-	2636514	79	-

Figure S4

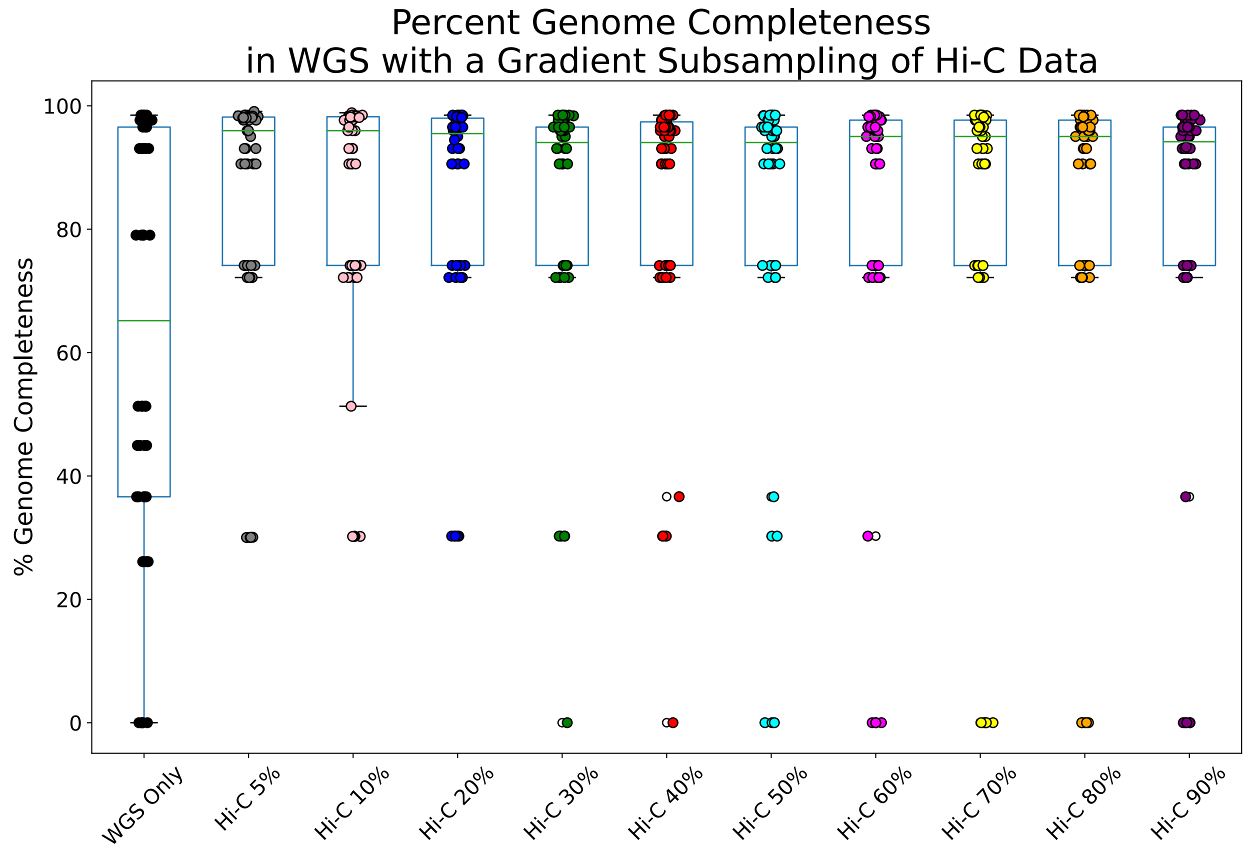

Figure S5

Genome completeness of Synthetic dataset using different Hi-C sequencing kits and laboratory protocols

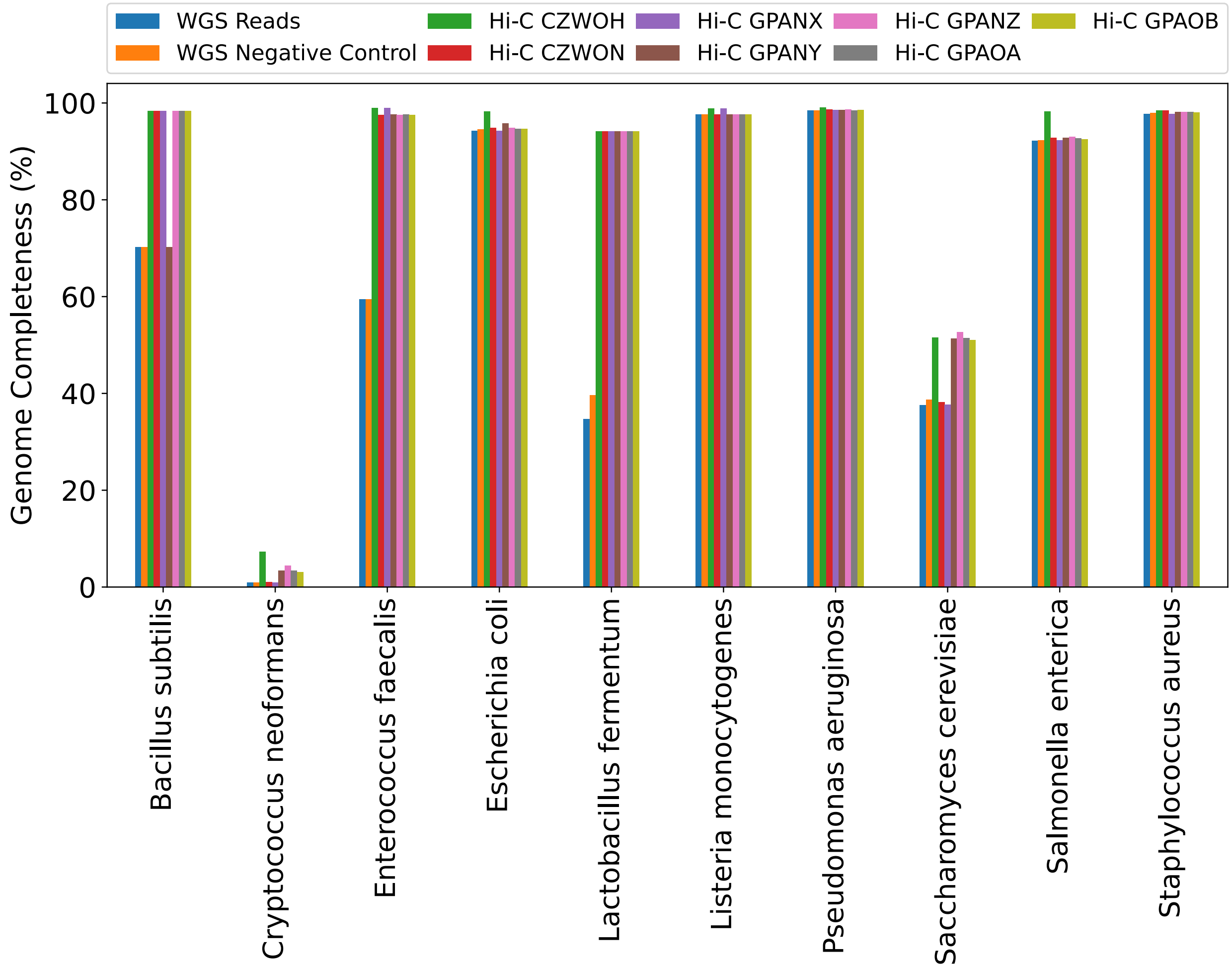

Figure S6

The following graphs shows the increase in genome completeness and contamination as the number of bins used for training increased in metaBAT-LR

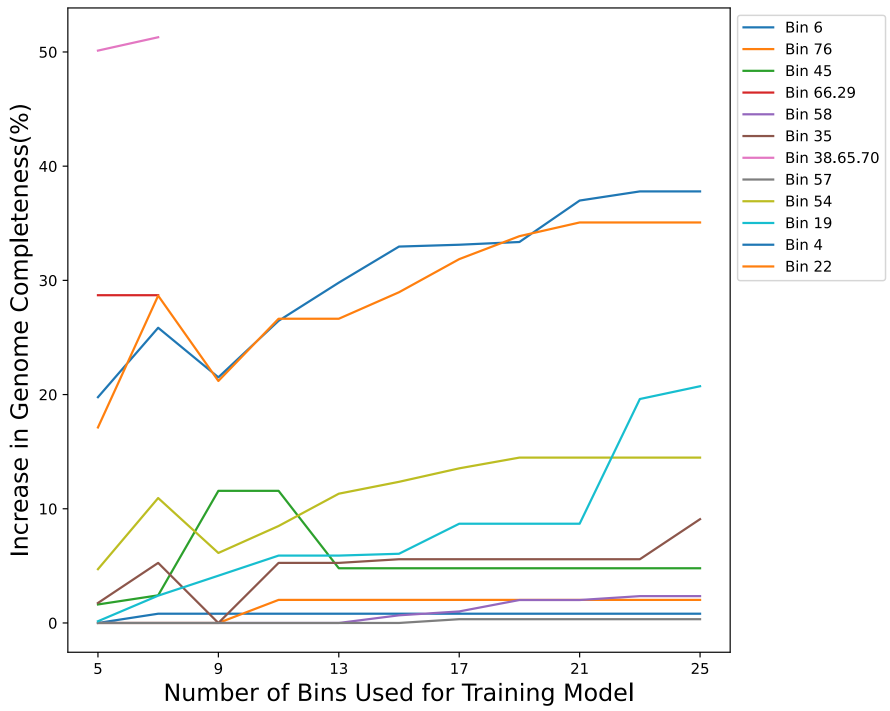

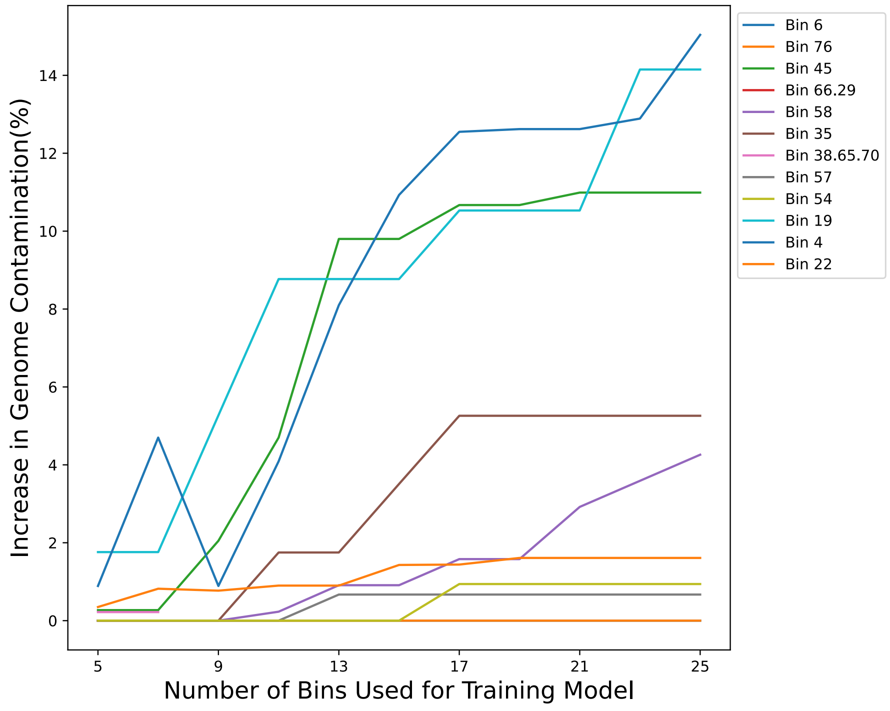

Figure S7

AMBER Metrics per Bin for Binning Results of MetaBAT-LR, Bin3C, ProxiMeta, BinSPreader, and HicSPAdes

Method	Most abundant genome	Purity (bp)	Completeness (bp)	Bin size (bp)	True positives (bp)	True size of most abundant genome (bp)
Metabat-LR	Cryptococcus_neoformans	0.614083	0.993729218	26908340	16523956	16628228
Metabat-LR	Pseudomonas_aeruginosa	1	0.992840772	6689917	6689917	6738157
Metabat-LR	Bacillus_subtilis	1	0.973731652	3845203	3845203	3948935
Metabat-LR	Salmonella_enterica	1	0.959432452	3801635	3801635	3962379
Metabat-LR	Listeria_monocytogenes	1	0.955797301	2923826	2923826	3059044
Metabat-LR	Staphylococcus_aureus	1	0.952857201	2636614	2636614	2767061
Metabat-LR	Escherichia_coli	0.976899	0.952648595	5353282	5229614	5489552
Metabat-LR	Enterococcus_faecalis	1	0.933150449	2577732	2577732	2762397
Metabat-LR	Lactobacillus_fermentum	1	0.766190951	1375135	1375135	1794768
Bin3C	Enterococcus_faecalis	1	0.241639243	471768	471768	1952365
Bin3C	Bacillus_subtilis	1	0.235613438	1018420	1018420	4322419
Bin3C	Enterococcus_faecalis	1	0.13480676	263192	263192	1952365
Bin3C	Escherichia_coli	1	0.122800875	328637	328637	2676178
Bin3C	Enterococcus_faecalis	1	0.091916214	179454	179454	1952365
ProxiMeta	Lactobacillus_fermentum	1	0.873480603	1784375	1784375	2042833
ProxiMeta	Enterococcus_faecalis	1	0.843231533	2814066	2814066	3337240
ProxiMeta	Escherichia_coli	1	0.726853448	4419990	4419990	6080992
ProxiMeta	Bacillus_subtilis	1	0.50339061	3975773	3975773	7897988
ProxiMeta	Bacillus_subtilis	1	0.267943684	2116216	2116216	7897988
ProxiMeta	Saccharomyces_cerevisiae	1	0.201275392	2200756	2200756	10934054
BinSPreader	Pseudomonas_aeruginosa	0.943307	0.999935155	7160065	6754139	6754577
BinSPreader	Escherichia_coli	0.927832	0.999804336	5931306	5503254	5504331
BinSPreader	Staphylococcus_aureus	0.950192	0.995406724	2832163	2691099	2703517
BinSPreader	Salmonella_enterica	0.994483	0.969410795	3840621	3819432	3939952
BinSPreader	Listeria_monocytogenes	0.996269	0.960242867	2941521	2930546	3051880
BinSPreader	Enterococcus_faecalis	0.980731	0.526703106	1494751	1465948	2783253
BinSPreader	Bacillus_subtilis	0.999702	0.474988175	1894548	1893984	3987434
BinSPreader	Saccharomyces_cerevisiae	0.999599	0.466353704	5031688	5029669	10785095
BinSPreader	Enterococcus_faecalis	0.999779	0.408737186	1137870	1137619	2783253
BinSPreader	Lactobacillus_fermentum	0.999518	0.334857753	606048	605756	1808995

BinSPreader	Bacillus_subtilis	1	0.28635057	1141804	1141804	3987434
BinSPreader	Lactobacillus_fermentum	0.992778	0.249168737	454024	450745	1808995
BinSPreader	Bacillus_subtilis	0.97707	0.22361298	912567	891642	3987434
BinSPreader	Lactobacillus_fermentum	1	0.194730223	352266	352266	1808995
BinSPreader	Lactobacillus_fermentum	1	0.158831838	287326	287326	1808995
HicSPAdes	Enterococcus_faecalis	1	0.977263906	2812330	2812330	2877759
HicSPAdes	Lactobacillus_fermentum	0.559051	0.970770699	3139131	1754935	1807775
HicSPAdes	Bacillus_subtilis	0.918259	0.722286837	4329686	3975773	5504424
HicSPAdes	Listeria_monocytogenes	1	0.69644852	2923826	2923826	4198194
HicSPAdes	Staphylococcus_aureus	1	0.685021292	2682666	2682666	3916179
HicSPAdes	Escherichia_coli	1	0.364142836	2159225	2159225	5929610
HicSPAdes	Salmonella_enterica	1	0.284700371	1090801	1090801	3831400
HicSPAdes	Escherichia_coli	1	0.25641366	1520433	1520433	5929610
HicSPAdes	Bacillus_subtilis	1	0.175765711	967489	967489	5504424
HicSPAdes	Escherichia_coli	1	0.130360175	772985	772985	5929610
HicSPAdes	Listeria_monocytogenes	1	0.115670691	485608	485608	4198194
HicSPAdes	Salmonella_enterica	1	0.103257034	395619	395619	3831400
HicSPAdes	Salmonella_enterica	1	0.084767448	324778	324778	3831400
HicSPAdes	Salmonella_enterica	0.551149	0.058441823	406268	223914	3831400
HicSPAdes	Salmonella_enterica	1	0.035684606	136722	136722	3831400
HicSPAdes	Escherichia_coli	1	0.035423915	210050	210050	5929610
HicSPAdes	Escherichia_coli	1	0.033652972	199549	199549	5929610
HicSPAdes	Salmonella_enterica	1	0.029753354	113997	113997	3831400
HicSPAdes	Salmonella_enterica	1	0.025604479	98101	98101	3831400
HicSPAdes	Escherichia_coli	1	0.021039664	124757	124757	5929610
HicSPAdes	Salmonella_enterica	1	0.016467871	63095	63095	3831400
HicSPAdes	Escherichia_coli	1	0.015066084	89336	89336	5929610
HicSPAdes	Escherichia_coli	1	0.006366186	37749	37749	5929610
HicSPAdes	Lactobacillus_fermentum	1	0.00622312	11250	11250	1807775
HicSPAdes	Cryptococcus_neoformans	1	0.004270272	71191	71191	16671302
HicSPAdes	Salmonella_enterica	1	0.003817143	14625	14625	3831400
HicSPAdes	Saccharomyces_cerevisiae	1	0.001999476	21760	21760	10882850
HicSPAdes	Escherichia_coli	1	0.001699775	10079	10079	5929610
HicSPAdes	Saccharomyces_cerevisiae	1	0.001620439	17635	17635	10882850
HicSPAdes	Saccharomyces_cerevisiae	1	0.001351576	14709	14709	10882850
HicSPAdes	Saccharomyces_cerevisiae	1	0.001271266	13835	13835	10882850
HicSPAdes	Saccharomyces_cerevisiae	1	0.000916672	9976	9976	10882850
HicSPAdes	Saccharomyces_cerevisiae	1	0.000756695	8235	8235	10882850
HicSPAdes	Saccharomyces_cerevisiae	1	0.000748885	8150	8150	10882850
HicSPAdes	Saccharomyces_cerevisiae	1	0.000744566	8103	8103	10882850

HicSPAdes	Saccharomyces_cerevisiae	1	0.000741258	8067	8067	10882850
HicSPAdes	Saccharomyces_cerevisiae	1	0.000710016	7727	7727	10882850
HicSPAdes	Saccharomyces_cerevisiae	1	0.000651392	7089	7089	10882850
HicSPAdes	Saccharomyces_cerevisiae	1	0.00062309	6781	6781	10882850
HicSPAdes	Saccharomyces_cerevisiae	1	0.000621436	6763	6763	10882850
HicSPAdes	Saccharomyces_cerevisiae	1	0.000620977	6758	6758	10882850
HicSPAdes	Saccharomyces_cerevisiae	1	0.000594789	6473	6473	10882850
HicSPAdes	Saccharomyces_cerevisiae	1	0.000588357	6403	6403	10882850
HicSPAdes	Cryptococcus_neoformans	1	0.000571881	9534	9534	16671302
HicSPAdes	Saccharomyces_cerevisiae	1	0.000525322	5717	5717	10882850
HicSPAdes	Saccharomyces_cerevisiae	1	0.000507863	5527	5527	10882850
HicSPAdes	Saccharomyces_cerevisiae	1	0.000498675	5427	5427	10882850
HicSPAdes	Saccharomyces_cerevisiae	1	0.000447677	4872	4872	10882850
HicSPAdes	Cryptococcus_neoformans	1	0.00028816	4804	4804	16671302

Figure S8

AMBER generated plots for binning results of MetaBAT-LR, Bin3C, ProxiMeta, BinSPreader, HicSPAdes, and Metabat2 with no Hi-C reads

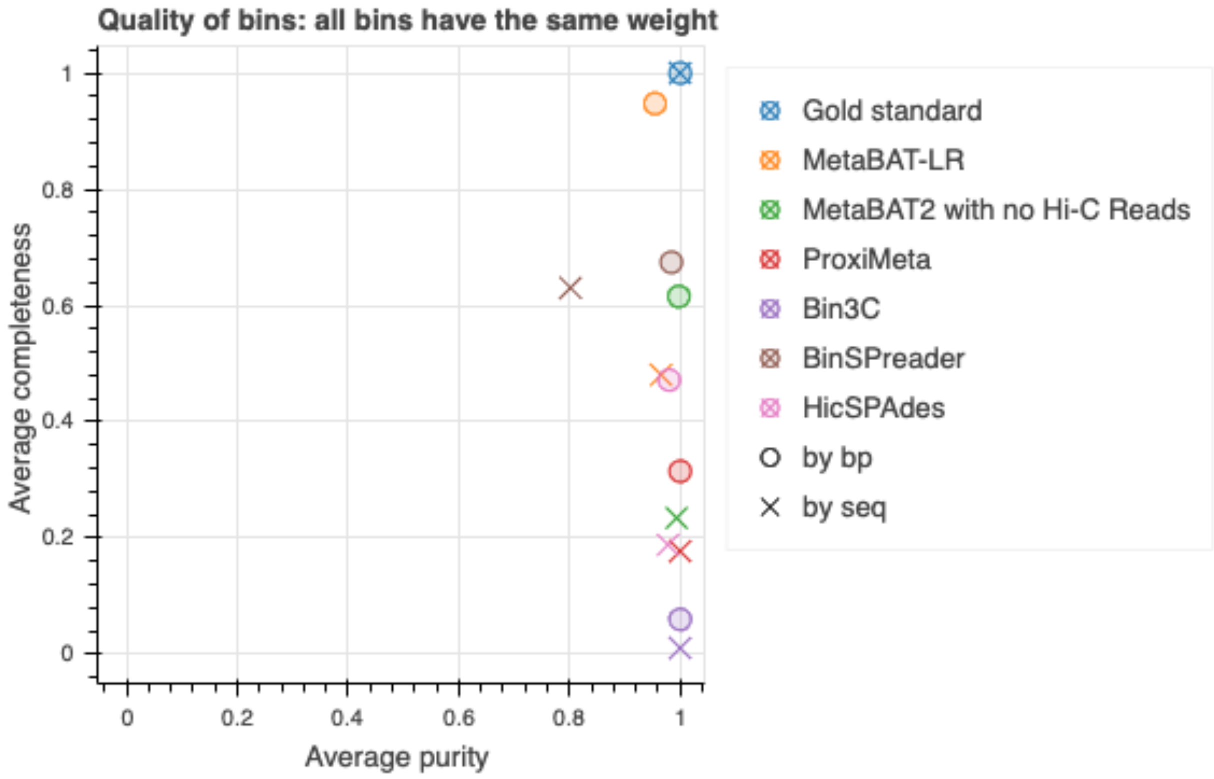

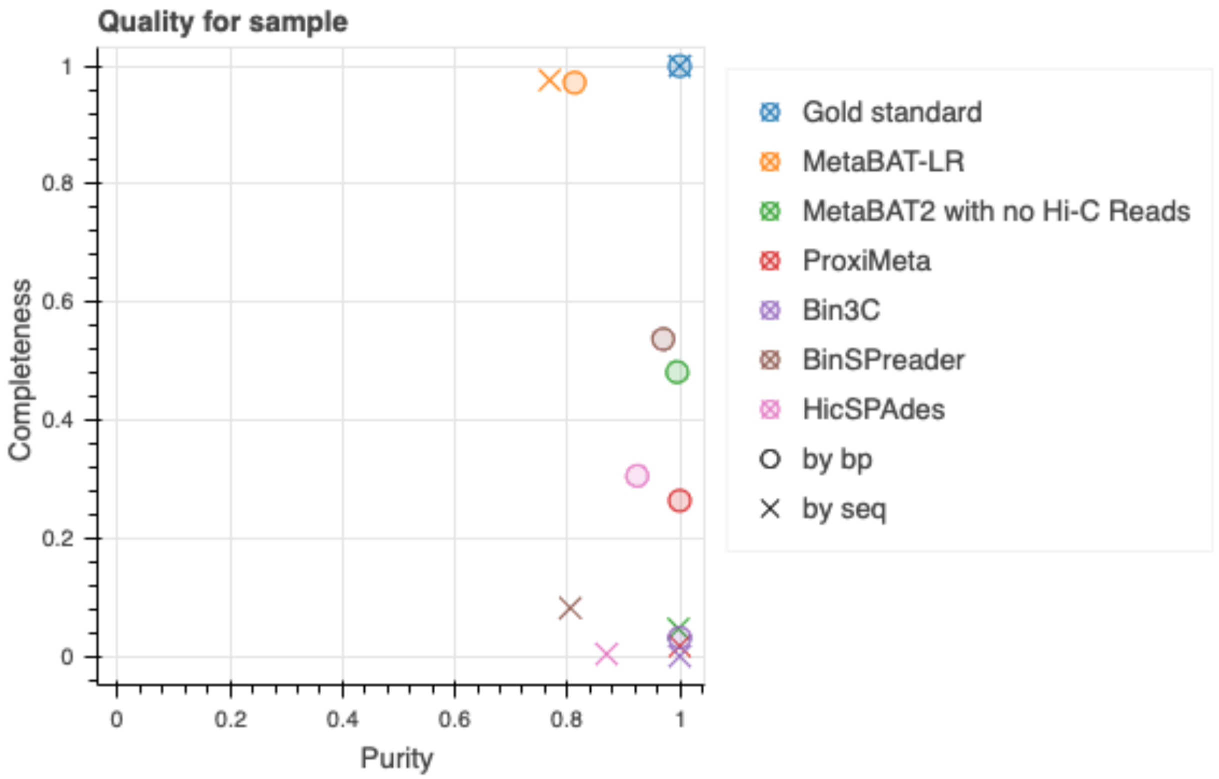
Quality for sample
Completeness
Purity
0
0.2
0.4
0.6
0.8
1
Gold standard
MetaBAT-LR
MetaBAT2 with no Hi-C Reads
ProxiMeta
Bin3C
BinSPreader
HicSPAdes
by bp
by seq

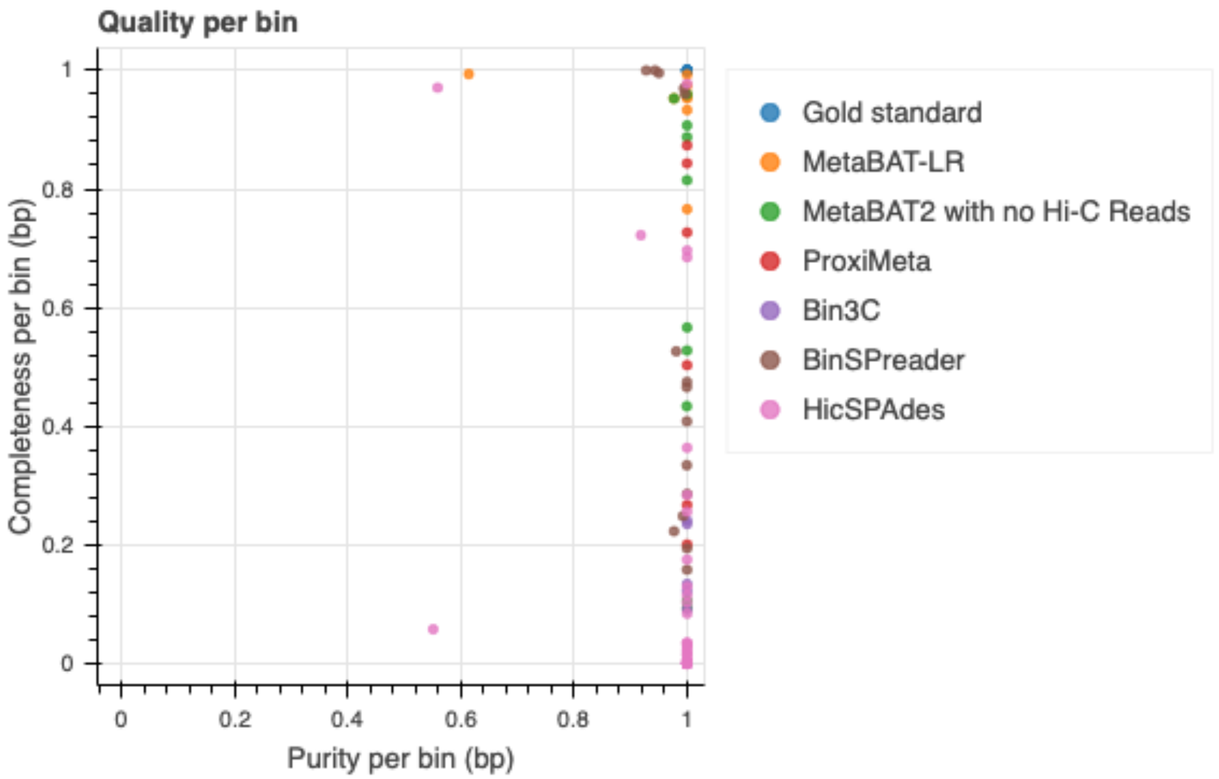
Quality per bin
Completeness per bin (bp)
Purity per bin (bp)
0
0.2
0.4
0.6
0.8
1
Gold standard
MetaBAT-LR
MetaBAT2 with no Hi-C Reads
ProxiMeta
Bin3C
BinSPreader
HicSPAdes

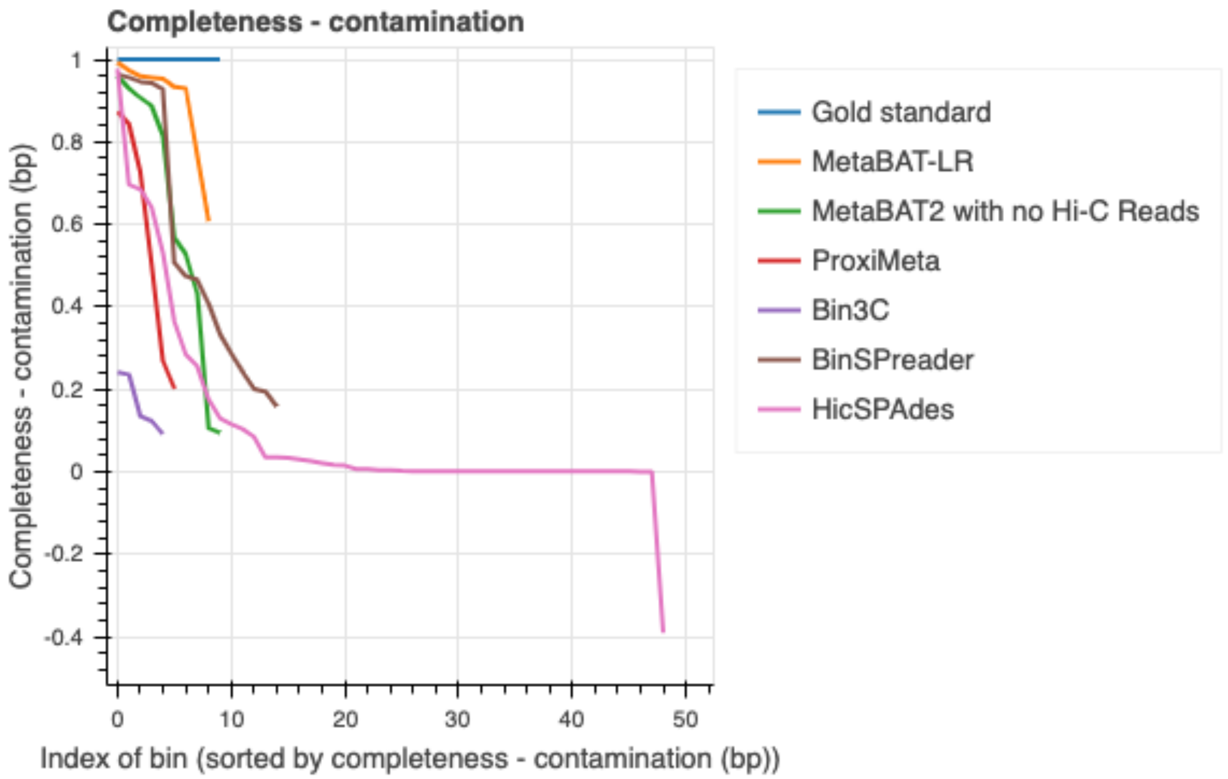
Completeness - contamination
1
0.8
0.6
0.4
0.2
0
-0.2
-0.4
Completeness - contamination (bp)
0
10
20
30
40
50
Index of bin (sorted by completeness - contamination (bp))
Gold standard
MetaBAT-LR
MetaBAT2 with no Hi-C Reads
ProxiMeta
Bin3C
BinSPreader
HicSPAdes

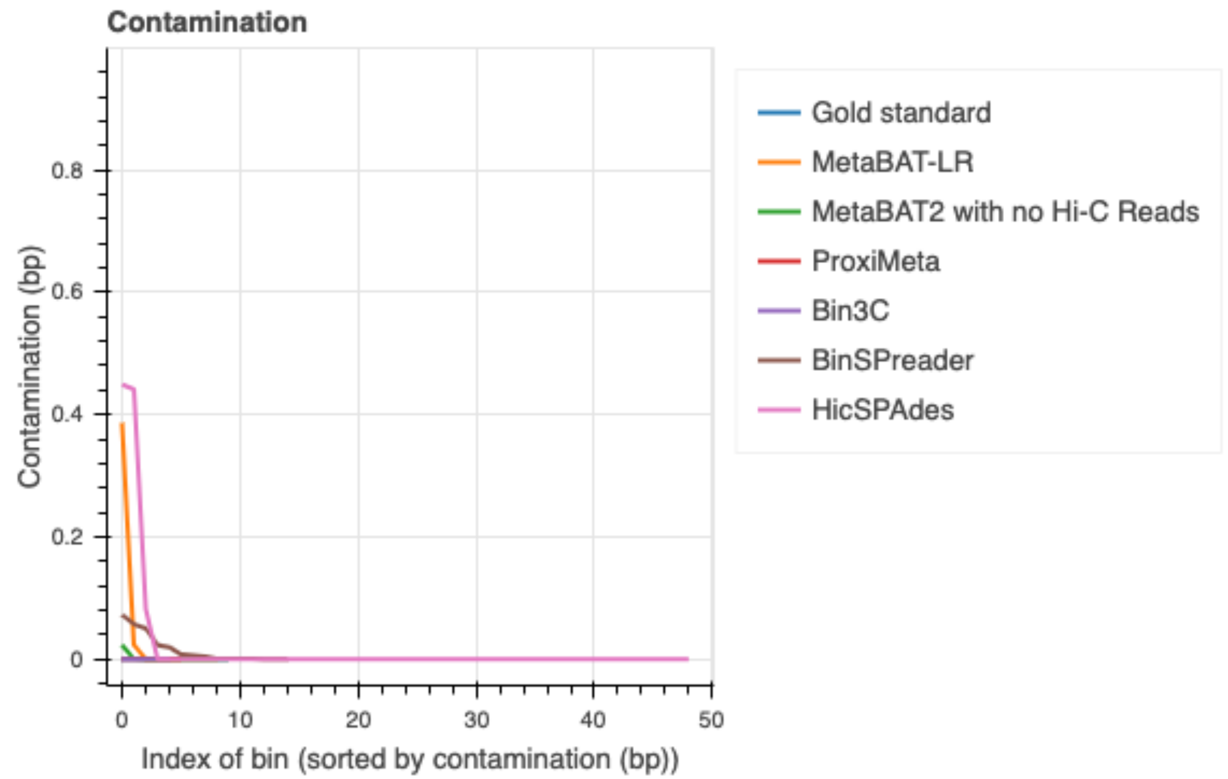
Contamination
0.8
0.6
0.4
0.2
0
Contamination (bp)
0
10
20
30
40
50
Index of bin (sorted by contamination (bp))
Gold standard
MetaBAT-LR
MetaBAT2 with no Hi-C Reads
ProxiMeta
Bin3C
BinSPreader
HicSPAdes

Figure S9

Metabat-LR Unique Bins from Human Fecal Dataset

Completeness	Contamination	Bin ID	Marker Lineage
96.31	0.00	Bin.15.fa	o__Bacteroidales (UID2654)
89.13	4.52	Bin.49.fa	f__Lachnospiraceae (UID1256)

Figure S10

The following charts show the number of metagenome bins found by each method at varying genome completeness levels. All bins are of medium quality or higher (having genome completeness of greater than or equal to 50% and contamination levels lower than 10%)

AMBER Output Comparing Completeness for the Zymo Mock Synthetic Dataset

	Completeness				
Method	50%-60%	60%-70%	70%-80%	80%-90%	>90%
MetaBAT-LR	0	0	1	0	7
ProxiMeta	1	0	1	2	0
BinSPreader	1	0	0	0	5
Bin3C	0	0	0	0	0
HicSPAdes	0	2	1	0	1

dRep Output Comparing Completeness for Cat Fecal Dataset

	Completeness				
Method	50%-60%	60%-70%	70%-80%	80%-90%	>90%
MetaBAT-LR	2	3	5	7	26
ProxiMeta	1	1	7	4	28
BinSPreader	3	4	5	4	18
Bin3C	1	3	4	5	23
HicSPAdes	2	2	3	2	21

dRep Output Comparing Completeness for Human Fecal Dataset

	Completeness				
Method	50%-60%	60%-70%	70%-80%	80%-90%	>90%
MetaBAT-LR	2	2	5	5	23
ProxiMeta	7	0	5	3	27
BinSPreader	3	2	4	3	14
Bin3C	1	1	3	1	28
HicSPAdes	2	2	3	2	19
